# Inflammasome-dependent Pyroptosis and IL-18 Protect against *Burkholderia pseudomallei* Lung Infection while IL-1β Is Deleterious

**DOI:** 10.1371/journal.ppat.1002452

**Published:** 2011-12-29

**Authors:** Ivonne Ceballos-Olvera, Manoranjan Sahoo, Mark A. Miller, Laura del Barrio, Fabio Re

**Affiliations:** Department of Microbiology, Immunology, and Biochemistry, University of Tennessee Health Science Center, Memphis, Tennessee, United States of America; University of Toronto, Canada

## Abstract

*Burkholderia pseudomallei* is a Gram-negative bacterium that infects macrophages and other cell types and causes melioidosis. The interaction of *B. pseudomallei* with the inflammasome and the role of pyroptosis, IL-1β, and IL-18 during melioidosis have not been investigated in detail. Here we show that the Nod-like receptors (NLR) NLRP3 and NLRC4 differentially regulate pyroptosis and production of IL-1β and IL-18 and are critical for inflammasome-mediated resistance to melioidosis. *In vitro* production of IL-1β by macrophages or dendritic cells infected with *B. pseudomallei* was dependent on NLRC4 and NLRP3 while pyroptosis required only NLRC4. Mice deficient in the inflammasome components ASC, caspase-1, NLRC4, and NLRP3, were dramatically more susceptible to lung infection with *B. pseudomallei* than WT mice. The heightened susceptibility of *Nlrp3^-/-^* mice was due to decreased production of IL-18 and IL-1β. In contrast, *Nlrc4^-/-^* mice produced IL-1β and IL-18 in higher amount than WT mice and their high susceptibility was due to decreased pyroptosis and consequently higher bacterial burdens. Analyses of IL-18-deficient mice revealed that IL-18 is essential for survival primarily because of its ability to induce IFNγ production. In contrast, studies using IL-1RI-deficient mice or WT mice treated with either IL-1β or IL-1 receptor agonist revealed that IL-1β has deleterious effects during melioidosis. The detrimental role of IL-1β appeared to be due, in part, to excessive recruitment of neutrophils to the lung. Because neutrophils do not express NLRC4 and therefore fail to undergo pyroptosis, they may be permissive to *B. pseudomallei* intracellular growth. Administration of neutrophil-recruitment inhibitors IL-1ra or the CXCR2 neutrophil chemokine receptor antagonist antileukinate protected *Nlrc4^-/-^* mice from lethal doses of *B. pseudomallei* and decreased systemic dissemination of bacteria. Thus, the NLRP3 and NLRC4 inflammasomes have non-redundant protective roles in melioidosis: NLRC4 regulates pyroptosis while NLRP3 regulates production of protective IL-18 and deleterious IL-1β.

## Author Summary

The disease melioidosis is caused by the intracellular bacterium *Burkholderia pseudomallei*, a potential bioterrorism agent. Here we examined the interaction of *B. pseudomallei* with the inflammasome, an important innate immune pathway that regulates at least two host responses protective against infections: 1) secretion of the proinflammatory cytokines IL-1β and IL-18 and 2) induction of pyroptosis, a form of cell death that restricts intracellular bacteria growth. Using a mouse model of melioidosis we show that two distinct inflammasomes are activated by *B. pseudomallei* infection. One, containing the Nod-like receptor (NLR) NLRP3, mediates IL-1β and IL-18 induction. The other contains a different NLR called NLRC4 and mediates pyroptosis. Pyroptosis and IL-18 production were equally important for resistance to *B. pseudomallei*. Surprisingly, IL-1β was found to be deleterious in melioidosis. The detrimental role of IL-1β during melioidosis was due, in part, to excessive recruitment of neutrophils to the lung. We show that neutrophils do not express NLRC4, fail to undergo pyroptosis, and, therefore, may be permissive to *B. pseudomallei* intracellular replication leading to increased bacterial burden and morbidity/mortality. Thus, the NLRP3 and NLRC4 inflammasomes have non-redundant protective roles in melioidosis: NLRC4 regulates pyroptosis while NLRP3 regulates production of protective IL-18 and deleterious IL-1β.

## Introduction

The ability to detect infection by pathogenic microbes and to restrict their growth are fundamental for the wellbeing of multicellular organisms. Pattern recognition receptors, including the Toll-like receptor (TLR) and the NLR, recognize microbial products and “danger signals” released by stressed cells and, in turn, activate signaling pathways that initiate the inflammatory response and regulate development of adaptive immunity. TLR are expressed on the cell surface or in endosomal compartments and their stimulation results in activation of the NF-κB, MAPK, and IRF signaling pathways culminating in transcriptional induction of a large number of genes. NLR, in contrast, are located in the cytoplasm, which they survey for evidence of danger or infection (reviewed in ref. [1]). Some NLR control activation of the inflammasome, a multiprotein complex that contains, in addition to a NLR, the adaptor molecule ASC and the protease caspase-1. Activation of caspase-1 in the context of the inflammasome is responsible for the proteolytic processing of the immature forms of IL-1β and IL-18, a modification required for the secretion and bio-activity of these proinflammatory cytokines. Activation of caspase-1 also triggers a form of cell death, known as pyroptosis, that effectively restricts intracellular bacterial growth [2], [3]. Production of IL-1β and IL-18 and induction of pyroptosis have been shown to be protective effector mechanisms against many infectious agents. NLRP3 and NLRC4 are the best characterized NLR molecules. NLRP3 controls caspase-1 activation in response to “danger signals”, several particles and crystals, and various bacteria, virus, and fungi. Although the logic that oversees the activation of the NLRP3 inflammasome is still elusive, it appears that disruption of cell membrane integrity may be a common event triggered by the NLRP3 activators. The NLRC4 inflammasome is responsive to a narrower spectrum of activators including cytoplasmically delivered bacterial flagellin and the basal rod constituent of various bacterial Type III secretion systems (T3SS). The T3SS apparatus is used by several bacteria, including *Salmonella, Yersinia, Pseudomonas, Shigella, Legionella, and Burkholderia* to inject virulence factors into the cytoplasm of target cells. Recent works demonstrated that the specificity of the mouse NLRC4 for flagellin or rod proteins is determined by its interaction with the NLR molecules NAIP5 or NAIP2, respectively [4], [5].

*Burkholderia pseudomallei* is a Gram-negative flagellate bacterium that causes melioidosis, a disease endemic to South-East Asia and other tropical regions [6], [7] and the most common cause of pneumonia-derived sepsis in Thailand. Because melioidosis carries a high fatality rate, *B. pseudomallei* is classified as category B potential bioterrorism agent by the Center for Disease Control and NIAID. *B. pseudomallei* infection can be contracted through ingestion, inhalation, or subcutaneous inoculation and leads to broad-spectrum disease forms including pneumonia, septicemia, and organ abscesses. Following infection of macrophages and other non-phagocytic cell types, *B. pseudomallei* is able to escape the phagosome and invade and replicate in the host cell cytoplasm, directly spreading from cell to cell using actin-tail propulsion. Macrophages and IFNγ have been shown to play a critical role in protection from melioidosis [8]–[10] and several *B. pseudomallei* virulence factors have been identified including the bacterial capsule [11], the lipopolysaccharide [12], and one of the three T3SS possessed by *B. pseudomallei* [13]. Analysis of mouse strains with different susceptibility to *B. pseudomallei* infection indicates that the early phases of the infection are crucial for survival [14], [15], emphasizing the necessity for better understanding of innate immune responses during melioidosis. With this goal in mind, using a murine model of melioidosis we have performed a detailed analysis of the role of the inflammasome components NLRP3, NLRC4, ASC, and caspase-1 and the effector mechanisms IL-1β, IL-18, and pyroptosis.

## Results

### NLRP3 and NLRC4 differentially regulate production of IL-1β and pyroptosis

**Figure 1:**
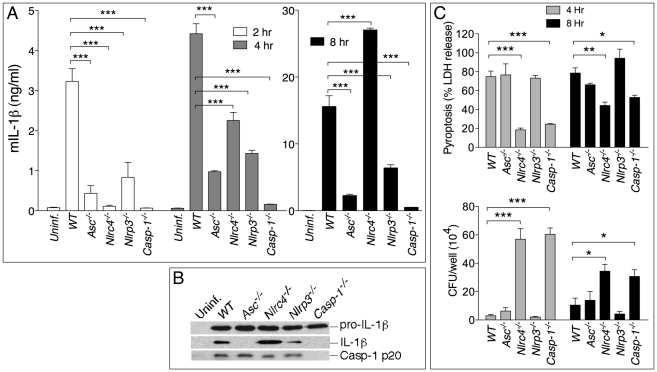
NLRP3 and NLRC4 differentially regulate production of IL-1β and pyroptosis. BMDM were infected with *B. pseudomallei* at MOI of 10. (A) Secretion of mature IL-1β was measured in conditioned supernatants of infected and uninfected cells at the indicated times. (B) Processing of IL-1β and caspase-1 was detected by immunoblot in 8 h conditioned supernatants from A. Pro-IL-1β was detected in cell lysate of the infected cells. (C) Induction of pyroptosis was measured as LDH release in conditioned supernatants of infected BMDM (MOI 10) (upper panel). Infected BMDM were lysed at the indicated time points after infection and intracellular bacteria growth was quantitated (lower panel). One experiment representative of three is shown. *p<0.05, **p<0.01, ***p<0.001 (1way ANOVA).

To identify the pathway responsible for IL-1β and IL-18 secretion in response to infection with *B. pseudomallei*, bone marrow-derived macrophages (BMDM) or dendritic cells (BMDC) derived from WT mice or mice deficient in the inflammasome components ASC, NLRP3, NLRC4, or caspase-1 were infected *in vitro* with *B. pseudomallei* and secretion of IL-1β in culture supernatants was measured. As shown in figure 1A, secretion of IL-1β by *Asc^-/-^, Nlrp3^-/-^,* and *Casp1* ^-/-^ BMDM was markedly reduced compared to WT BMDM. Production of IL-1β during the first hours of the infection was also significantly reduced in *Nlrc4^-/-^* cells. However, later in the infection process (8 hours) *Nlrc4^-/-^* cells secreted IL-1β at levels considerably higher than WT cells. Secretion of IL-18 followed a similar pattern (data not shown). Immunoblotting of the supernatants confirmed processing of IL-1β and of caspase-1 to the mature 17 kDa and p20 forms, respectively (figure 1B). Interestingly, although caspase-1 was activated in *Asc^-/-^* cells, processing and secretion of IL-1β was not observed. NLRC4 possesses an amino-terminal CARD domain that can recruit and activate caspase-1 independently of ASC. It is unclear at present why activation of caspase-1 in *Asc^-/-^* cells is not sufficient to trigger secretion of mature IL-1β, a phenomenon previously reported by other groups [16]. The differences in IL-1β and IL-18 secretion were observed regardless of the number of bacteria used to infect cells (MOI 10, 50, or 100, data not shown) and were not due to differential induction of pro-IL-1β, which was present at comparable amounts in all the cell lysates. Thus, the NLRC4 and NLRP3 inflammasomes are both mediating release of IL-1β and IL-18 by myeloid cells infected with *B. pseudomallei*.

Inflammasome-mediated induction of pyroptosis has been demonstrated to be a mechanism that restricts growth of certain intracellular bacteria [2], [3]. To measure induction of pyroptosis in cells infected with *B. pseudomallei* we used a kanamycin protection assay that allows only replication of intracellular bacteria whereas cells that undergo pyroptosis expose the bacteria to the microbicidal action of the antibiotic present in the medium. Induction of pyroptosis and intracellular bacterial replication were measured in WT or inflammasome-deficient BMDM infected with *B. pseudomallei*. As shown in figure 1C (upper graph), pyroptosis of infected cells (as measured by release of LDH in culture supernatants) was significantly reduced in *Casp1^-/-^* and *Nlrc4^-/-^* cells compared to WT and *Nlrp3^-/-^*. Importantly, induction of pyroptosis was not lost in *Asc^-/-^* cells. NLRC4-mediated pyroptosis induced by other bacteria is also reported to be ASC-independent [16]–[19]. Consistent with the role of pyroptosis as a mechanism to restrict intracellular bacteria growth, considerably less intracellular bacteria were recovered from WT, *Nlrp3^-/-^,* and *Asc^-/-^* cells than *Casp1^-/-^* or *Nlrc4^-/-^* cells at all time points (figure 1C, lower graph). Similar results regarding IL-1β processing and secretion and induction of pyroptosis were obtained using BMDC derived from the inflammasome–deficient mice (supplementary figure S1).

Taken together these results show that infection of macrophages and dendritic cells with *B. pseudomallei* leads to activation of the NLRC4 and NLRP3 inflammasomes. NLRC4 contributes to IL-1β during the early phase of the infection and induction of pyroptosis that restricts bacterial growth. NLRP3 does not control pyroptosis and primarily controls IL-1β secretion. It should be noted that the defective IL-1β production of *Nlrc4^-/-^* and *Nlrp3^-/-^* cells cannot be ascribed to the difference in induction of pyroptosis: thus *Nlrp3^-/-^* cells produce less cytokine than WT cells despite undergoing pyroptosis to the same extent as WT cells. Conversely, *Nlrc4^-/-^* cells, which are resistant to pyroptosis, still produce less cytokine than WT cells at the early time point. However, at later time points *Nlrc4^-/-^* cells produce considerably more IL-1β than WT cells. This is likely due to the fact that WT cells rapidly die after infection while *Nlrc4^-/-^* cells remain viable and continue to synthesize and secrete IL-1β.

### Role of inflammasomes in murine melioidosis

**Figure 2:**
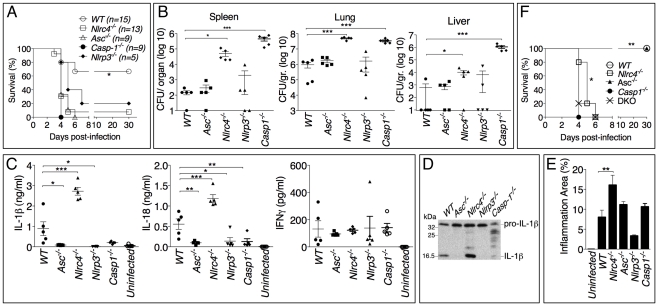
Differential contributions of NLRP3 and NLRC4 to melioidosis. (A) Mice were intranasally infected with *B. pseudomallei* (100 CFU) and their survival was monitored. *p<0.05 (Kaplan-Meier), WT compared to other genotypes (B) Mice were sacrificed 48 hours post-infection and the bacterial burdens were measured in organ homogenates. (C) Cytokines were measured in BALF obtained 48 hours post-infection. (D) Processing of IL-1β was detected by immunoblot in BALF from C. (E) Lung sections were stained with H&E and the total area of the inflammatory nodules was measured and expressed as percentage of the total lung lobe area. *p<0.05, **p<0.01, ***p<0.001 (1way ANOVA). (F) Mice were intranasaly infected with *B. pseudomallei* (25 CFU) and their survival was monitored. **p<0.01 WT compared to other genotypes and *p<0.05 *Casp-1^-/-^* compared to *Nlrc4^-/-^* (Kaplan-Meier).

The role of the inflammasome during *in vivo B. pseudomallei* infection was next analyzed using a mouse model of melioidosis (figure 2). WT mice or inflammasome-deficient mice were infected intranasally with *B. pseudomallei* (100 CFU) and their weight (not shown) and survival were monitored (figure 2A). All mice started to lose weight 2 days post-infection. Generally, mice that survived the infection started to recover weight 7 days post-infection. *Casp1^-/-^*, *Nlrc4^-/-^*, and *Asc^-/-^* mice were extremely susceptible to melioidosis compared to WT mice. *Nlrp3^-/-^* mice were also considerably more susceptible than WT mice but slightly more resistant than the other inflammasome deficient mice. Measurement of the bacterial burdens in lungs, spleens, and livers of infected mice 24 hours (data not shown) and 48 hours post-infection revealed that *Nlrc4^-/-^* and *Casp1* ^-/-^ mice carried considerably higher burdens in all three organs than WT mice (figure 2B). Surprisingly, the bacterial burden of *Asc^-/-^* and *Nlrp3^-/-^* mice was not significantly different from that of WT mice at the tested time points despite their higher mortality.

Cytokine levels were measured in bronchio-alveolar lavage fluids (BALF) obtained from infected mice (figure 2C). Confirming the *in vitro* results, IL-1β and IL-18 levels were severely reduced in *Asc^-/-^*, *Casp1* ^-/-^ and *Nlrp3^-/-^* mice. In contrast, IL-1β and IL-18 were present in the lungs of *Nlrc4^-/-^* mice in amounts considerably higher than WT mice. Immunoblotting experiments confirmed that the IL-1β measured by ELISA was in fact the p17 mature form of IL-1β (figure 2D). Thus, although the *in vitro* experiments demonstrated that both the NLRP3 and the NLRC4 inflammasome contribute to IL-1β and IL-18 production in response to *B. pseudomallei* infection, it is the NLRP3 inflammasome that primarily mediates production of these cytokines *in vivo*. The levels of several other proinflammatory cytokines, including IL-1α (figure S2), were significantly elevated in *Nlrc4^-/-^* BALF. It is interesting to note that the levels of IL-18 in BALF of *Asc^-/-^* and *Casp1^-/-^* mice, although very low, were higher than uninfected mice suggesting the existence of inflammasome-independent mechanisms to produce IL-1β and IL-18, as it has been previously shown in models of highly neutrophilic inflammation [20]-[23].

Histological analysis of the infected lungs revealed extensive inflammatory cell infiltration in the lung parenchyma (data not shown). The area of the inflammatory nodules, relative to the total area of the lung lobe, was calculated for each given section and found to be significantly greater in *Nlrc4^-/-^* mice compared to WT mice (figure 2E). This result was consistent with the elevated levels of inflammatory cytokines and chemokines produced by *Nlrc4^-/-^* mice. Taken together these results suggest a scenario where failure of *Nlrc4^-/-^* infected macrophages to undergo pyroptosis results in higher bacterial burden and continued production of IL-1β and other factors that attract more inflammatory cells, perpetuating lung inflammation and promoting bacteria dissemination.

Thus, our results identified two distinct infammasome-mediated mechanisms that efficiently restrict *B. pseudomallei* growth and pathogenesis: production of the cytokines IL-1β and IL-18 and induction of pyroptosis. The high susceptibility of *Nlrp3^-/-^* and *Asc^-/-^* mice to meliodiosis is due to defective cytokine production while that of the *Nlrc4^-/-^* mice likely results from defective pyroptosis. *Casp1^-/-^* mice are impaired in both inflammasome effector mechanisms and, therefore, we predicted that they would be more vulnerable to *B. pseudomallei* than *Asc^-/-^* or *Il1-r1^-/-^-Il-18^-/-^* double knock-out mice (DKO) (that are defective in cytokines but retain pyroptosis) or *Nlrc4^-/-^* mice (that retain IL-1β/IL-18 functionality but are deficient in pyroptosis). This prediction was found to be correct. As shown in figure 2F, when mice were infected with only 25 CFU (a non-lethal dose for WT mice) the mean time to death of *Nlrc4^-/-^* and *Il-1r1^-/-^*-*Il-18^-/-^* DKO mice was slightly but significantly (*p*<0.05, Kaplan-Meier test) increased compared to *Casp1^-/-^* mice. Surprisingly, *Asc^-/-^* mice, which should be equivalent to DKO because of the absence of IL-1β or IL-18, survived the infection. This may be explained by the observation that IL-18, although drastically reduced, it is still detectable in *Asc^-/-^* mice at higher level than uninfected mice (figure 2C).

### Role of IL-18 and IL-1β in murine melioidosis

**Figure 3:**
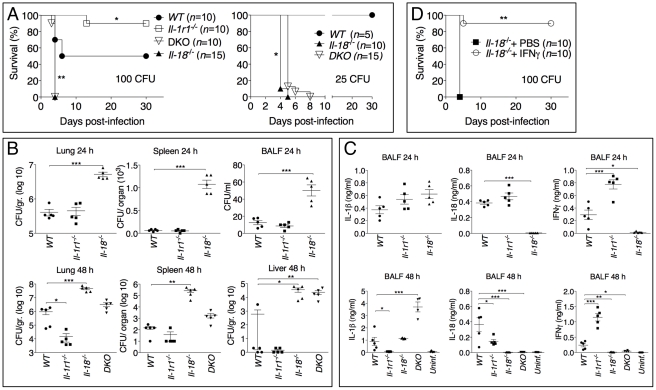
Differential contributions of IL-1 and IL-18 to melioidosis. (A) Mice were intranasaly infected with *B. pseudomallei* (100 CFU, left, or 25 CFU, right) and their survival was monitored. *p<0.05, **p<0.01 (Kaplan-Meier), WT compared to other genotypes (left), *Il-18^-/-^* compared to DKO (right). (B, C) Mice infected with *B. pseudomallei* (100 CFU) were sacrificed 24 hours or 48 hours postinfection and the bacterial burdens in organ homogenates and BALF (B) or cytokines levels in BALF (C) were measured. *p<0.05, **p<0.01, ***p<0.001 (1way ANOVA). (D) *Il-18^-/-^* mice were intranasally infected with *B. pseudomallei* (100 CFU) and their survival monitored. Mice were administered daily injections of PBS or IFNγ (1 µg) for the first 8 days. **p<0.01 (Kaplan-Meier).

We next analyzed the role of the inflammasome-dependent cytokines IL-1β and IL-18 during murine melioidosis. IL-18-deficient mice were extremely susceptible to *B. pseudomallei* infection even when infected with 25 CFU, a dose of bacteria that caused no mortality and only mild weight loss in WT mice (figure 3A). In contrast, *Il-1r1^-/-^* mice displayed increased resistance to *B. pseudomallei* infection compared to WT mice (figure 3A and see below). The survival of mice deficient in both IL-18 and IL-1RI (DKO) was indistinguishable from the *Il-18^-/-^* mice when the animals were infected with 100 CFU. However, in DKO mice infected with 25 CFU (figure 3A, right panel) the concomitant absence of IL-18 and IL-1RI provided a significant advantage over *Il-18^-/-^* mice (*p*<0.05) suggesting a detrimental role of IL-1RI-mediated signaling in melioidosis (see below).

Confirming the different susceptibilities of *Il-18^-/-^* and *Il-1r1^-/-^* mice to melioidosis, the bacterial burdens observed in the lungs, spleens, livers, and BALF of infected *Il-18^-/-^* mice were dramatically higher than that of WT mice even at early time points (24 hours post infection, figure 3B). In contrast, significantly lower amounts of bacteria were recovered 48 hours post infection from *Il-1r1^-/-^* mice compared to WT mice confirming their higher resistance.

**Figure 4:**
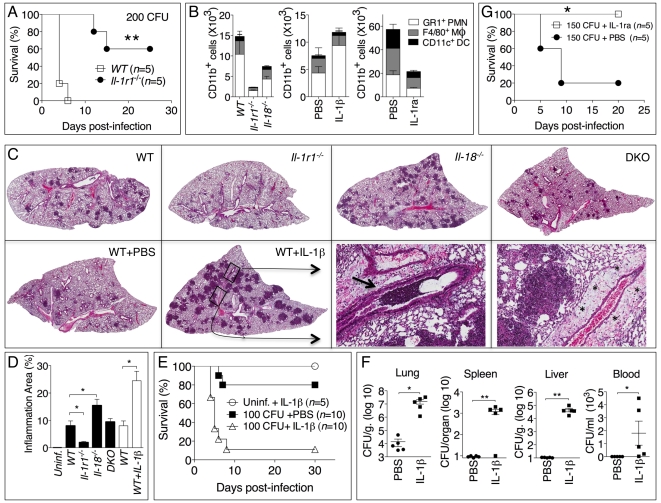
Deleterious role of IL-1β in melioidosis. (A) Mice were infected intranasally with *B. pseudomallei* (200 CFU) and their survival was monitored. (B) Flow cytometric analysis for myeloid cell composition of BALF obtained from the indicated infected mouse strains at 24 h (left); infected WT mice injected with IL-1β at 48 h (center graph); or infected WT mice injected with PBS or IL-1ra at 72 h. *p<0.05, ***p<0.001 (1way ANOVA). (C) Histopathology of lungs of infected mice of indicated genotype (0.8X magnification) at 48 h post-infection (upper row) or WT mice injected with PBS or IL-1β at 72 hrs post-infection (lower row). Bottom right panel shows 10X magnification of the indicated insets from WT+IL-1β showing airways obstruction (arrow head) and perivascular edema (asterisks). One image representative of five animals/experimental group. (D) The total area of the inflammatory nodules of lung sections of C was measured and expressed as percentage of the total lung lobe area. *p<0.05 (*t*-test). (E) WT mice were infected intranasally with *B. pseudomallei* (100 CFU) and received daily i.p. injections of PBS or IL-1β (1 µg). One group of mice were treated with IL-1β but not infected. **p<0.01, ***p<0.001 (Kaplan-Meyer). (F) Mice infected and IL-1β-injected as in E were sacrificed 72 h post-infection and the bacterial burden was measured in organs and blood. *p<0.05, **p<0.01 (1way ANOVA). (G) WT mice were infected intranasally with *B. pseudomallei* (150 CFU) and were administered daily injections of PBS or IL-1ra. *p<0.05 (Kaplan-Meyer).

Measurements of cytokines in the BALF obtained from mice at 24 and 48 hours post-infection (figure 3C) indicated that the levels of IFNγ were drastically reduced in *Il-18^-/-^* mice, a finding consistent with the established function of IL-18 as an IFNγ-inducing cytokine. Remarkably, IFNγ levels in *Il-1r1^-/-^* mice were greatly increased compared to WT mice. The levels of the neutrophil attractants Mip-2, KC, and IL-17 were also decreased in *Il-1r1^-/-^* mice and increased in *Il-18^-/-^* mice (figure S2). The number of inflammatory cells recovered from the BALF of infected *Il-1r1^-/-^* mice was significantly decreased compared to WT mice (see figure 4B, left panel). Histological analysis of the infected lungs revealed extensive inflammatory cell infiltration in the lung parenchyma of *Il-18^-/-^* mice (see figure 4C). The area of the inflammatory nodules, relative to the total area of the lung lobe, was calculated for each given section and found to be significantly greater in *Il-18^-/-^* mice compared to WT mice (figure 4D).

Considering that IFNγ is known to play a protective role during several bacterial infections, including *B. pseudomallei* [8]–[10], these results suggested that the reduced resistance of *Il-18^-/-^* mice to *B. pseudomallei* infection may be due to lack of IFNγ induction. To test this hypothesis, a group of *Il-18^-/-^* mice infected with *B. pseudomallei* were given daily intraperitoneal injections of either recombinant IFNγ or PBS. As shown in figure 3D, exogenous IFNγ completely protected the mice suggesting that IL-18 exerts its protective action primarily through induction of IFNγ.

### Deleterious role of IL-1β in melioidosis

The results of figure 3 showed that *Il-1r1^-/-^* mice were more resistant to lung infection with *B. pseudomallei*. This appeared even more evident when mice were infected with higher doses of *B. pseudomallei* that killed all WT mice but only a fraction of the *Il-1r1^-/-^* mice (figure 4A). Recruitment of neutrophils, macrophages, and dendritic cells into alveolar spaces was decreased in *Il-1r1^-/-^* mice compared to WT mice (figure 4B, left graph). Lower levels of the neutrophil enzyme myeloperoxidase (MPO) were detected in the BALF of *Il-1r1^-/-^* mice compared to WT (figure S3). The extent of lung inflammation, as measured by the number and size of inflammatory nodules, was also significantly decreased in *Il-1r1^-/-^* mice (figure 4C, and 4D).

To further test the hypothesis that IL-1R-mediated signaling has a deleterious role in this model of melioidosis, WT mice were infected with 100 CFU *B. pseudomallei* and were given daily intraperitoneal injections of IL-1β or PBS (figure 4E). All mice that received the cytokine succumbed to the infection compared to significantly higher survival of the control group. Injection of IL-1β in non-infected mice had no deleterious effect aside from a transient, negligible weight loss (not shown). The bacteria burdens in organs of IL-1β-treated mice 72 hours post infection were dramatically higher than the control group and bacteremia was detected in IL-1β-treated mice but not control mice (figure 4F). Higher number of neutrophils, macrophages, and dendritic cells were found in the BALF of IL-1β-treated mice (figure 4B, center graph). This correlated with increased level of MPO in BALF (figure S3). The increased inflammatory cell recruitment to the lungs of IL-1β-treated mice was likely due to the induction, by IL-1β, of neutrophil chemoattractans KC (CXCL1) and MIP-2 (CXCL-2), which in fact were detected at very high levels in the BALF of IL-1β-treated mice (figure S2). Histological analysis of lung sections of mice treated with IL-1β showed a dramatic increase in the number and size of the foci of infiltrating inflammatory cells (figure 4C, lower left panels) and evidence of perivascular edema and airway obstruction (figure 4C, lower right panels).

If IL-1β in fact has a detrimental effect during melioidosis, inhibition of its activity should lower morbidity and mortality of mice infected with *B. pseudomallei.* As shown in figure 4G, administration of the IL-1 receptor antagonist IL-1ra protected mice from infection with lethal doses of *B. pseudomallei.* Mice treated with IL-1ra had decreased recruitment of inflammatory cells to the alveolar spaces (figure 4B, right graph) lower level of MPO in BALF (figure S3), and less severe lung pathology (data not shown).

### Neutrophils fail to restrict *B. pseudomallei* intracellular growth and are resistant to pyroptosis

**Figure 5:**
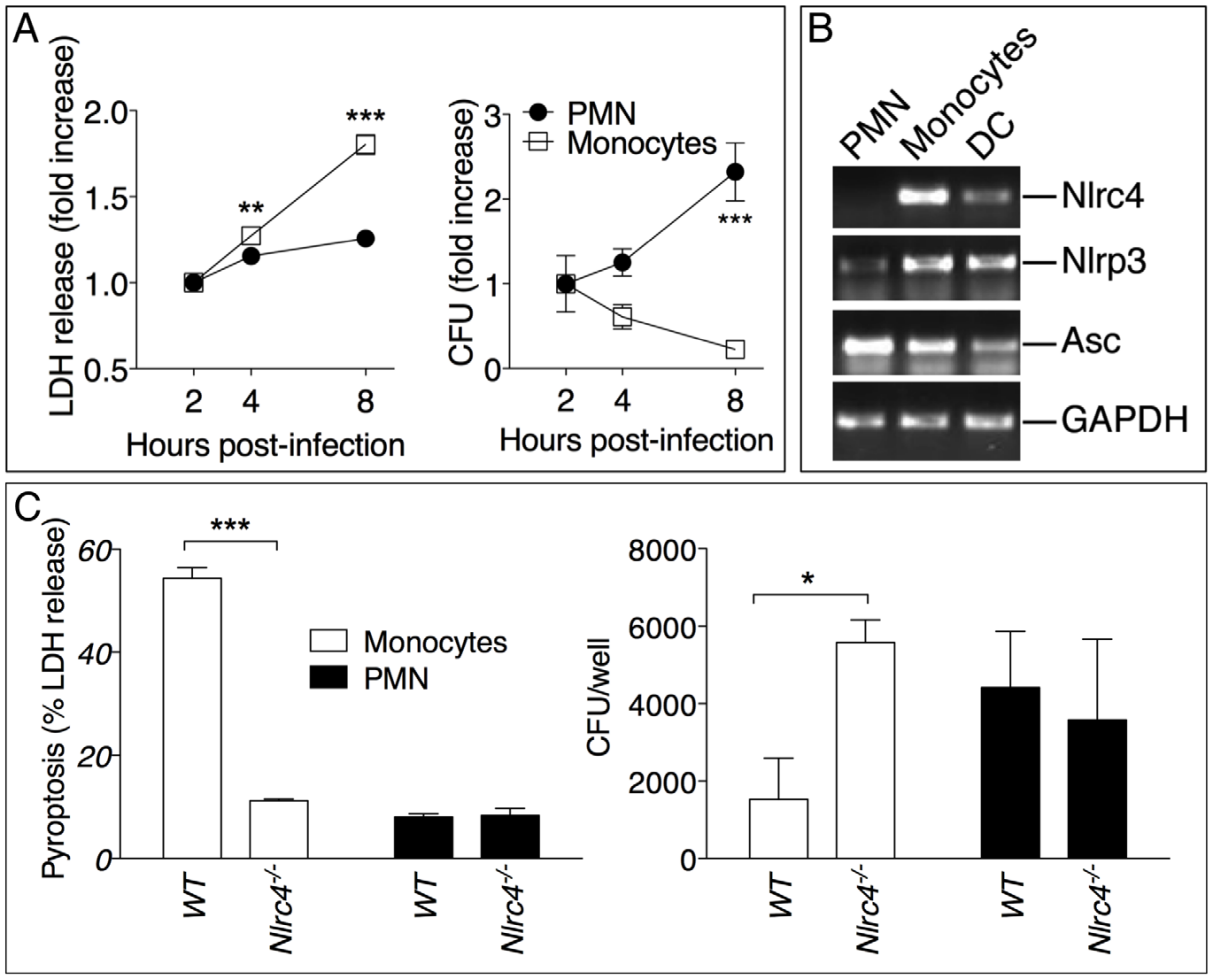
Neutrophils do not undergo pyroptosis and fail to restrict *B. pseudomallei* replication. (A) Human neutrophils or monocytes were infected with *B. pseudomallei* (MOI 50) and pyroptosis and intracellular bacterial growth were measured at the indicated time points. One experiment representative of two is shown. Data are shown as the fold increase normalized to the 2 h values. (B) RT-PCR analysis of total RNA from the indicated cell types. (C) WT or *Nlrc4^-/-^* mouse neutrophils and monocytic cells were infected with *B. pseudomallei* (MOI 50) and pyroptosis and intracellular bacterial growth were measured 8 hours post infection. * *p*<0.05, **p<0.01, ***p<0.001 (2way ANOVA, post-test Bonferroni).

**Figure 6:**
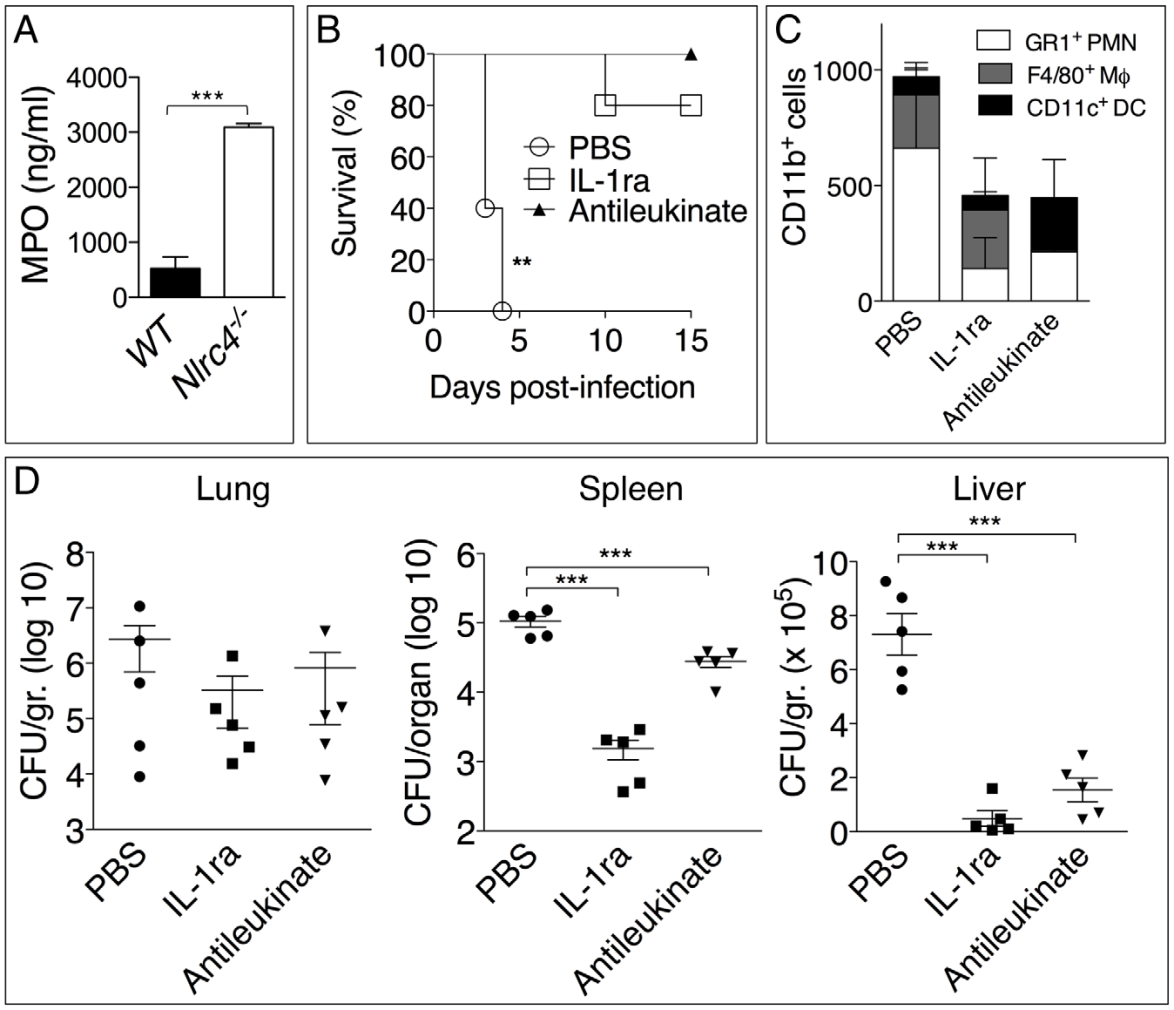
Inhibition of neutrophil recruitment to the lung protects *Nlrc4^-/-^* mice from melioidosis. (A) Myeloperoxidase was measured in BALF of WT and *Nlrc4^-/-^* mice. (B) *Nlrc4^-/-^* mice were infected intranasally with *B. pseudomallei* (25 CFU) and were administered daily injections of PBS or IL-1ra (i.p.) or antileukinate (s.c.). **p<0.01, ***p<0.001 (Kaplan-Meyer). (C) Flow cytometric analysis for myeloid cell composition of BALF obtained from *Nlrc4^-/-^* mice infected and treated with IL-1ra or antileukinate. (D) *Nlrc4^-/-^* mice infected and treated with IL-1ra or antileukinate were sacrificed 48 h post-infection and the bacterial burden was measured in organs. *p<0.05, **p<0.01 (1way ANOVA).

Surprisingly, in our experiments lower numbers of neutrophils in *Il-1r1^-/-^* mice correlated with lower bacterial burdens while IL-1β administration resulted in increased neutrophil recruitment *but also* increased bacterial burdens and systemic dissemination. These results would be consistent with the notion that neutrophils are not very effective at containing *B. pseudomallei* infection and, in fact, may foster its spread. In support of this idea, human neutrophils infected with *B. pseudomallei* underwent pyroptosis at a much slower rate than infected monocytes (figure 5A). Concomitantly, intracellular bacteria growth increased with time in infected neutrophils but decreased in monocytes. Consistent with previously published results [3], neutrophils did not express NLRC4 mRNA (figure 5B) suggesting they may be resistant to pyroptosis. Similar results were obtained using neutrophils and CD11b^+^ monocytic cells isolated from mouse bone marrow (figure 5C). WT monocytes infected with *B. pseudomallei* underwent pyroptosis and failed to support bacteria replication whereas *Nlrc4^-/-^* cells were resistant to pyropotosis and supported *B. pseudomallei* intracellular replication. In contrast, both WT and *Nlrc4^-/-^* neutrophils did not undergo pyroptosis and supported *B. pseudomallei* intracellular replication to the same extent. These results suggest that the deleterious role of IL-1β during melioidosis may be due, in part, to excessive recruitment of neutrophils, a cell type that may be permissive for *B. pseudomallei* replication. We decided to test this hypothesis in *Nlrc4^-/-^* mice. As shown in figure 2E, infected *Nlrc4^-/-^* mice showed a significantly higher degree of lung inflammation. Consistent with higher neutrophil influx in the lung of *Nlrc4^-/-^* mice, the levels of the neutrophil enzyme MPO were significantly increased in their BALF compared to WT mice (figure 6A). To test the hypothesis that excessive neutrophil influx is deleterious during melioidosis, *Nlrc4^-/-^* mice were injected with IL-1ra or with antileukinate, a hexapeptide that acts as a CXCR2 neutrophil chemokine receptor antagonist. Both factors have been shown to inhibit neutrophil recruitment to inflammatory sites in different animal models including lung inflammation [24]–[26]. As shown in figure 6B, administration of IL-1ra or antileukinate protected *Nlrc4^-/-^* mice infected with low doses of *B. pseudomallei*. The number of inflammatory cells in the BALF of *Nlrc4^-/-^* mice treated with IL-1ra or antileukinate was reduced compared to mice who received PBS injection (figure 6C) and lower levels of MPO were detected in the BALF of injected mice (figure S3). Moreover, systemic spread of bacteria to spleen or liver was reduced by administration of either drug (figure 6D).

## Discussion

**Figure 7:**
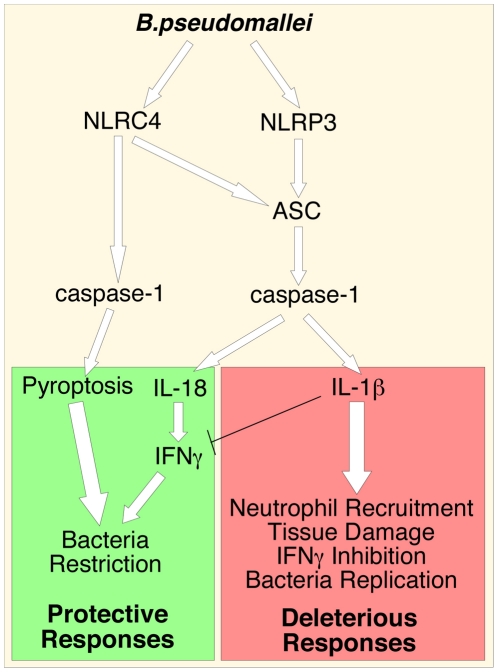
Inflammasomes-dependent protective and deleterious responses activated by *B. pseudomallei.* * B. pseudomallei* induces NLRC4-dependent pyroptosis that restricts intracellular bacterial growth. Activation of NLRP3-inflammasome leads to production of IL-18 and IL-1β. IL-18 is protective because of its induction of IFNγ. IL-1β deleterious role may be due to several reasons including excessive recruitment of neutrophils, which may support intracellular growth of *B. pseudomallei*, tissue damage, and inhibition of IFNγ production.

The inflammatory response to infection consists of several protective effector mechanisms that must be activated and orchestrated in order to maximize microbicidal functions and stimulation of adaptive immunity while, at the same time, minimize damage to the host tissues. Alteration in this balance may result in excessive and non-resolving inflammation that leads to severe morbidity and mortality [27]. It is becoming clear that to be effective but non-pathogenic the inflammatory response must be tailored to each specific pathogen. Here we have analyzed the role of a very important inflammatory pathway during infection with the lung pathogen *B. pseudomallei*. Using a murine model of melioidosis we have determined the role of various components of the inflammasome and the downstream effector mechanisms (production of IL-1β, IL-18, and pyroptosis) and we report several novel discoveries that greatly increase our understanding of the pathogenesis of melioidosis (see model in figure 7).

First, we found that both NLRC4 and NLRP3 play non-redundant roles during detection of *B. pseudomallei*. Analysis of *in vitro* infected macrophages or dendritic cells allowed us to estimate the relative contribution of NLRC4 and NLRP3 to IL-1β production. Our findings indicated that production of IL-1β is primarily dependent on the NLRP3 inflammasome. During the early phase of the infection the NLRC4 inflammasome also significantly contributes to IL-1β production. We posit that this pattern likely reflects the fact that the NLRC4 inflammasome responds to T3SS deployment, which occurs early in the infection cycle, while activation of NLRP3 may require escape from the phagosome, which is a relatively slower event [28]. *B. pseudomallei* , including the strain used in our study, possesses at least three T3SS gene clusters, one of which is similar to the *Salmonella* SP-1 pathogenicity island and has been shown to be an important virulence factor required for escape from the phagosome, induction of IL-1β production, and pathogenicity [13], [28]. In addition to mediating host recognition of cytosol-delivered flagellin, NLRC4 also recognizes a structural motif found in the basal body rod components of the T3SS of various bacteria, including *B. pseudomallei* [29]. We have determined (data not shown) that transfection of *B. pseudomallei* flagellin protein into the cytoplasm of BMDC leads to NLRC4-dependent production of IL-1β. This result agrees with previously published evidence and indicates that *B. pseudomallei* (like some other bacteria) expresses multiple factors (e.g. flagellin, basal rods) that are recognized by the NLRC4 inflammasome. The mechanism responsible for NLRP3 activation by *B. pseudomallei* remains unclear.

In addition to controlling IL-1β and IL-18 production, NLRC4 also mediates pyroptosis, a form of cell death that is an effective mechanism to restrict growth and dissemination of intracellular bacteria [3]. Here we showed that *B. pseudomallei*-induced pyroptosis was caspase-1-dependent but ASC-independent, in agreement with works that showed ASC redundancy for pyroptosis induced by other bacteria [16]–[19]. However, the fact that production of IL-1β in response to *B. pseudomallei* infection is seriously compromised in *Asc^-/-^* cells indicates that this adaptor molecule plays a critical role in NLRC4-mediated cytokine production and suggests that NLRC4 can form two distinct inflammasomes: one that contains ASC and regulates IL-1β processing, and one devoid of ASC that activates caspase-1 and triggers pyroptosis, as recently proposed [30]. It has been recently shown [4], [5] that NAIP molecules determine the specificity of NLRC4 for its activators and, we would further speculate, for its down-stream effector mechanisms. Whether NLRC4 relies on other molecules to recognize *B. pseudomallei* remains to be ascertained. We tested the susceptibility to *B. pseudomallei* of C57BL/6J-Chr13^A/J^/NaJ mice, a consomic C57BL/6 strain that carries the A/J NAIP5 allele that renders them susceptible *to Legionella* infection [31]-[34], and found that they were indistinguishable from WT mice (data not shown).

Analysis of inflammasomes-deficient mice intranasaly infected with *B. pseudomallei* confirmed the importance of ASC, caspase-1, and both NLRP3 and NLRC4 inflammasomes for resistance to melioidosis. However, quite surprisingly, although production of IL-1β and IL-18 *in vitro* is mediated by both NLRP3 and NLRC4, *in vivo* it is exclusively dependent on the NLRP3-ASC-caspase-1 inflammasome (figure 2C). In contrast, *Nlrc4^-/-^* mice produce these cytokines in amounts that exceed even those detected in WT mice. Remarkably, despite the abundance of IL-1β and IL-18, *Nlrc4^-/-^* mice were dramatically more susceptible to melioidosis than WT mice, rapidly succumbed to the infection, and had very high organ's bacteria burden and worst neutrophilic lung inflammation. Thus, the critical role of NLRC4 during melioidosis is independent of IL-1β and IL-18 production. Rather, our results suggest that pyroptosis, which we show is defective in *Nlrc4^-/-^* cells, is a critical NLRC4 effector mechanism to fight *B. pseudomallei* and, in its absence, bacterial replication and IL-1β production proceeds unrestrained causing severe inflammation, morbidity and mortality. Moreover, our analysis indicates that pyroptosis and IL-18 are both required and contribute equally to resistance to melioidosis. Thus, deficiency of either is equally lethal while deficiency of both (*Casp1* ^-/-^ mice) further worsens the outcome. It is important to emphasize that our study is the first to demonstrate the importance of pyroptosis in the context of an infection with a clinically relevant human pathogen that has not been genetically manipulated, as opposed to the previous seminal work by Miao et al. [3] that elegantly employed genetically manipulated bacteria and mouse strains to identify pyroptosis as an effective innate immune defence mechanism against bacterial infections. Previous reports have demonstrated activation of both NLRP3 and NLRC4 inflammasomes in response to infection with *Legionella pneumophila* [16], *Listeria monocytogenes* [35], and *Salmonella typhimurium* [36]. However, in those infection models NLRP3 and NLRC4 appeared to play redundant roles while in our model we were able to assign distinct functions to each inflammasome.

A great number of publications have documented the role of IL-18 and IL-1β during infections with a variety of pathogens. Almost invariably, both cytokines were found to have a protective function. Remarkably, our results show that while *Il-18^-/-^* mice are profoundly vulnerable to melioidosis, as previously shown [37], *Il-1r1^-/-^* mice were unexpectedly more resistant than WT mice. The protective role of IL-18 during melioidosis appears to be related to its ability to induce IFNγ, as administration of exogenous IFNγ completely rescued the survival of *Il-18^-/-^* infected mice. IFNγ activates the microbicidal activity of macrophages and has been shown to be important for resistance against infection with many pathogens including *B. pseudomallei* [8]–[10]. It is interesting and surprising to see that *Asc^-/-^* and *Nlrp3^-/-^* mice, which are defective in IL-1β and IL-18 production, are more resistant to *B. pseudomallei* than mice lacking IL-18. It is worth noting that although IL-18 production is drastically reduced in *Asc^-/-^* and *Nlrp3^-/-^* mice, it is still detectable in these mice at higher level than uninfected mice. It is conceivable that this inflammasome-independent production of IL-18 may be sufficient to provide some level of protection to *Asc^-/-^* and *Nlrp3^-/-^* mice against infection with low *B. pseudomallei* CFU.

Our discovery that *Il-1r1^-/-^* mice were more resistant than WT to *B. pseudomallei* infection is quite surprising considering that this cytokine has been shown to be protective in several bacterial, viral, and fungal infection models [38]. Studies in humans have also shown that inhibition of the function of IL-1 using the IL-1R antagonist IL-1ra (Kineret) is associated with increased susceptibility to bacterial infection. Infected *Il-1r1^-/-^* mice had lower BALF levels of proinflammatory cytokines as well as a reduction of neutrophil influx into the lungs, bacterial burdens, and lung pathology. Consistent with a deleterious role of IL-1β in melioidosis, administration of recombinant IL-1β drastically increased mortality, inflammation, pathology, and bacteria burdens while administration of IL-1ra (Kineret) rescued the survival of WT mice infected with a lethal dose of *B. pseudomallei*.

The reason for the detrimental effect of IL-1β during melioidosis is unclear and it is likely that several factors determine this outcome. IL-1β is one of the most powerful proinflammatory cytokines, it affects virtually every organ, and several human pathologies are primarily driven by unrestrained IL-1β production. One possible mechanism to account for IL-1β's deleterious role in melioidosis may be related to its ability to inhibit IFNγ production through the induction of the cycloxigenase COX-2 and release of prostaglandin PGE_2_ [39], [40]. Our observation that the level of IFNγ, a protective factor against *B. pseudomallei,* was significantly higher in infected *Il-1r1^-/-^* than WT mice (figure 3C) supports this type of scenario in melioidosis. Interestingly IL-8, a potent neutrophil chemoattractant, was shown to enhance the intracellular growth and survival of *B. cepacia* in bronchial epithelial cell lines [41]. Whether IL-1β promotes *B. pseudomallei* intracellular replication is not known but our preliminary results indicated that induction of pyroptosis by *B. pseudomallei* was not affected by IL-1β.

IL-1β regulates neutrophil recruitment to inflammatory sites through multiple mechanisms including induction of KC, MIP-2, and IL-17, inflammatory mediators whose expression in our experiments correlated with the presence/absence of IL-1RI-mediated signaling. Excessive PMN recruitment is known to cause tissue damage leading to functional impairment of multiple organs, including the lungs [42], [43]. One of the most remarkable observations reported here is that the absence of IL-1 signaling was associated with reduced lung neutrophilic inflammation *but also* lower bacterial burdens in the lungs (figure 3B, 4B). Conversely, IL-1β administration resulted in increased neutrophil recruitment *but also* increased bacterial burdens and systemic dissemination. These results would be consistent with the idea that neutrophils are not very effective at containing *B. pseudomallei* infection and, in fact, may foster its spread despite their strong microbicidal activities. This notion is supported by our observation that human or mouse neutrophils infected with *B. pseudomallei* failed to undergo pyropotosis (figure 5), consistent with the finding that neutrophils do not express NLRC4 [3]. At the same time, intracellular *B. pseudomallei* replication proceeded unaffected in both WT and *Nlrc4^-/-^* neutrophils in agreement with a report that showed that *B. pseudomallei* is intrinsically resistant to killing by infected PMN [44]. In support for a deleterious role of neutrophils in melioidosis we found that inhibition of their recruitment by administration of IL-1ra or the CXCR2 neutrophil chemokine receptor antagonist antileukinate protected *Nlrc4^-/-^* mice from infection with low doses of *B. pseudomallei* and decreased systemic spread of bacteria (figure 6).

Taken together our results suggest the following scenario: failure of *Nlrc4^-/-^* infected macrophages to undergo pyroptosis results in higher bacteria burden and continued production of IL-1β and other factors that attract more inflammatory cells, including neutrophils, perpetuating excessive lung inflammation and promoting bacteria dissemination. It is tempting to speculate that IL-1β promotes *B. pseudomallei* growth possibly by increasing the local pool of infectable permissive cells, including PMN. Our conclusion that neutrophils are a permissive cell type for *B. pseudomallei* replication seems to contrast with a report [45] that indicated that depletion of neutrophils resulted in severe increase in mortality in a model of murine melioidosis. However, caution should be used in the interpretation of these types of experiments because systemic depletion of neutrophils devoids the host not only of their microbicidal function but also of the many immunomodulatory functions these cells exert [46]. Of note, mice deficient in osteopontin, a pleiotropic cytokine that is chemotactic for neutrophils, were shown to be more resistant to *B. pseudomallei* infection [47], supporting our conclusion that neutrophils have a detrimental role in melioidosis.

The notion that excessive inflammation may be detrimental in certain infection models is well accepted. For example, TLR-mediated signaling negatively affects the outcome of infections with West Nile Virus [48] or influenza virus [49]. The fact that IL-1β is deleterious in melioidosis but protective against other lung pathogens like *Klebsiella, Francisella, Mycobacterium*, Respiratory Syncytial Virus, and influenza virus likely reflects differences between the virulence strategy of *B. pseudomallei* and those other pathogens. The intensity, kinetics, and quality of the inflammatory response elicited by *B. pseudomallei* and its ability to suppress the induction of anti-inflammatory circuitries are phenomena that we are interested to investigate in detail. Despite an extensive literature search we could identify only a single report [26] where IL-1β was shown to be deleterious in bacterial infections. It was demonstrated that this cytokine had a negative effect on bacterial clearance in a model of pneumonia caused by *Pseudomonas aeruginosa*, an organism that shares features with *Burkholderia*, which was in fact previously classified in the *Pseudomonas* genus. Surprisingly, the same group also reported a deleterious role for IL-18 in this type of infection [50], a further indication that each pathogen displays unique virulence strategies. It has been shown that activation of the inflammasome exacerbates inflammation without restricting bacterial growth in a model of *Mycobacterial* infection [51]. That report did not examine the role of IL-1β but other work showed it is protective during tuberculosis [23].

This is the first report that has analyzed in detail the role of the inflammasome during melioidosis. Previous work has implicated caspase-1 [52] and IL-18 [37] in this infectious disease although the pathways that led to their activation were not investigated. Other species of *Burkholderia* have been used as model organisms to study aspects of inflammasome biology. Surprisingly, *B. thailandensis*, which is has been used as a model for melioidosis although it rarely causes disease in humans, was reported to cause similar disease in WT and IL-18- IL-1β-double deficient mice [3] suggesting that species of *Burkholderia* other than *B. pseudomallei* may not be reliable models for melioidosis.

In summary, our work shows that NLRP3 and NLRC4 play non-redundant roles during *B. pseudomallei* infection by differentially regulating pyroptosis and production of IL-1β and IL-18; it demonstrates that pyroptosis is an efficient effector mechanism to restrict *in vivo* bacterial growth and dissemination; it identifies a deleterious role of IL-1β in melioidosis possibly due to excessive recruitment of neutrophils, a cell type that may be permissive to replication of *B. pseudomallei*; and, finally, it indicates that inhibition of IL-1RI-mediated signaling may be a beneficial therapeutical approach for the treatment of melioidosis.

## Materials and Methods

### Ethics statement

All the animal experiments described in the present study were conducted in strict accordance with the recommendations in the *Guide for the Care and Use of Laboratory Animals* of the National Institutes of Health. All animal studies were conducted under a protocol approved by the University of Tennessee Health Science Center (UTHSC) Institutional Animal Care and Use Committee (IACUC, protocol #1854). All efforts were made to minimize suffering and ensure the highest ethical and humane standards. Research involving human blood is exempt from the human subjects regulations. Human neutrophils and monocytes were isolated from healthy donors Leukopacks obtained from Lifeblood Mid-South Regional Blood Center, Memphis TN. All leukopaks are obtained anonymously. The gender, race, and age of each donor are unknown to the investigators.

### Mice

C57BL/6, *Il-1r1^-/-^*, *Il-18^-/-^,* C57BL/6J-Chr13^A/J^/NaJ mice were purchased from Jackson lab. *Il-18^-/-^-Il-1r1^-/-^* double deficient mice (DKO) were obtained by crossing the parental single knock-out mice. *Asc^-/-^, Nlrp3* ^-/-^, *Nlrc4^-/-^* (from Vishva Dixit, Genentec) and *Casp1* ^-/-^ (from Fayyaz Sutterwala) were bred in our facility. All mouse strains were on C57BL/6 genetic background and were bred under specific pathogen-free conditions. Age-(8–12 weeks old) and sex-matched animals were used in all experiments. Generally, experimental groups were composed of at least 5 mice. Animal and *in vitro* experiments involving *B. pseudomallei* were performed under biosafety level 3 conditions in accordance with standard operating procedures approved by the Regional Biocontainment Laboratory at UTHSC.

### Bacteria, mice infection, and treatments

For all experiment the *B. pseudomallei* 1026b strain (a clinical virulent isolate) was used. Bacteria were grown in Luria broth (Difco) to mid-logarithmic phase, their titer was determined by plating serial dilutions on LB agar, and stocks were maintained frozen at −80°C in 20% glycerol. No loss in viability was observed over prolonged storage. For infections, frozen stocks were diluted in sterile PBS to the desired titer. Aliquots were plated on LB agar to confirm actual CFU. Mice were anesthetized with isoflurane using a Surgivet apparatus and 50 µl of bacteria suspension were applied to the nares. In some experiments, mice were injected i.p. daily with recombinant mouse IL-1β (1 µg) or IFNγ (1 µg). IL-1ra (Biovitrum) was administered by alternating s.c. and i.p. injections every 12 hours (60 mg/kg body weight). Antileukinate (American Peptide Company) was administered by s.c. injection (8 mg/kg body weight).

### Generation of mouse BMDM and BMDC

Mouse macrophages or dendritic cells were generated by incubating bone marrow cells in RPMI 1640-10%FCS supplemented with either rmM-CSF or rmGM-CSF (20 ng/ml) for 8 days, respectively.

### Isolation of mouse neutrophils

Neutrophils and monocytic cells were isolated from the bone marrow cells of WT or *Nlrc4^-/-^* mice using Miltenyi Ly6G microbeads. Flow-through cells, consisting mostly of monocytic cells, were further purified using Miltenyi CD11b microbeads.

### Isolation of human neutrophils and monocytes

Human neutrophils and monocytes were isolated from healthy donors Leukopacks obtained from Lifeblood Mid-South Regional Blood Center, Memphis TN. Blood was mixed with Isolymph (CTL Scientific Supply Corp.) (5∶1 ratio) and RBC were allowed to sediment for 60 min at RT. The leukocytes-enriched supernatant was washed, resuspended in PBS, and stratified over Isolymph cushion and centrifuged at 1,350 rpm for 40 min. The cell pellet containing RBC and neutrophils was treated with 0.2% NaCl for 30 seconds to lyse RBC and immediatedly treated with an equal volume of 1.6% NaCl. The PBMC containing ring from the Isolymph centrifugation step was collected, washed, and monocytes were purified using CD14 microbeads (Miltenyi). The procedure routinely yield populations of purity greater than 95%.

### Pyroptosis and intracellular bacteria growth (kanamycin protection assay)

Release of LDH in tissue culture media, a reflection of pyroptosis, was measured using the Roche Cytotox detection kit. BMDM, PMN, or monocytes (5×10^5^ cells) were plated in 24 well plates. Bacteria at different MOI were added to the cell culture and the plates were centrifuged at 1500 rpm for 10 minutes to maximize and synchronize infection and incubated for 30 minutes at 37°C. Cells were washed with PBS to remove extracellular bacteria and medium containing kanamycin (200 µg/ml) was added to inhibit extracellular bacteria growth. Media were collected at 1, 2, 4, 8, 12 hours post infection for LDH measurement. Cells were lysed in PBS-2% saponin-15% BSA and serial dilutions of the lysates were plated on LB agar plates containing streptomycin (100 µg/ml) using the Eddy Jet Spiral Plater (Neutec). Bacterial colonies were counted 48 hours later using the Flash & Grow Automated Bacterial Colony Counter (Neutec).

### Determination of bacteria growth in tissue culture and organs

Organs aseptically collected were weighted and homogenized in 1 ml PBS using 1 mm zirconium beads and the Mini16 bead beater. Serial dilutions were plated as described above.

### Western blot

Conditioned supernatants were separated by 12% PAGE electrophoresis, transferred to PVDF membranes, and probed with rabbit anti-caspase-1 (Upstate Biotechnologies) or goat anti-mIL-1β (R&D Systems).

### BALF collection and cytokines measurements

BALF were collected from euthanized mice by intratracheal injection and aspiration of 1 ml PBS. Cytokines levels in tissue culture conditioned supernatants and BALF were measured using the Milliplex mouse cytokine/chemokine panel (Millipore) and confirmed by ELISA using the following paired antibodies kits: mIL-1β and mIFNγ (eBioscience), mIL-18 (MBL Nagoya, Japan). MPO level in BALF were measured using the HyCult Biotech ELISA kit.

### Flow cytometry

Cells obtained from BALF were counted and stained with CD45, CD11b, CD11c, F4/80, GR1 (Ly6G), and analyzed with a LSRII BD flow cytometer.

### Histology and measurement of area of inflammatory foci

Formalin-fixed paraffin-embedded lung sections were stained with H&E and scanned using the Aperio Scanscope XT. The Aperio ImageScope software was used to quantitate the area of the inflammatory foci compared to the total lung lobe area. Results from lungs from 5 animals per group were combined.

### RT-PCR

Total RNA was extracted using Trizol (Invitrogen) and 100 ng were amplified (27 cycles) using Superscript III One-step RT-PCR (Invitrogen) and primers specific for human Nlrc4, Nlrp3, Asc, and GAPDH (primers' sequence available upon request).

### Statistical analysis

All data were expressed as mean ± S.E.M. Survival curves were compared using the log rank Kaplan-Meier test. 1way ANOVA and Tukey Post-test was used for analysis of the rest of data unless specified in the figure legends. Significance was set at *p*<0.05. Statistical analyses were performed using the GraphPad Prism 5.0.

### Accession numbers

UniProtKB/Swiss-Prot ID: IL-1β, P10749; IL-1R1, P13504; IL-18, P70380; NLRP3, Q8R4B8; NLRC4, Q3UP24; ASC, Q9EPB4; Casp-1, P29452; NAIP5, Q8CGT2.

## Supporting Information

Figure S1 **NLRP3 and NLRC4 differentially regulate production of IL-1β and IL-18 and pyroptosis.** BMDC were infected with *B. pseudomallei* at MOI of 10. (A) Secretion of mature IL-1β was measured in conditioned supernatants at the indicated times. (B) Processing of IL-1β and caspase-1 were detected by immunoblot in 8h conditioned supernatants from A. (C) BMDC infected with *B. pseudomallei* (MOI 10) were lysed at the indicated time points after infection and intracellular bacterial growth was quantitated (upper panel). Induction of pyroptosis was measured as LDH release in conditioned supernatants (lower panel). One experiment representative of four (A) or three (C) is shown. *p<0.05, **p<0.01, ***p<0.001 (1way ANOVA).
(TIF)

Figure S2 **Cytokines and chemokines were measured in BALF obtained from the indicated mouse strains 48 hours or 72 hours post-infection, as shown.** *p<0.05, **p<0.01, ***p<0.001 (1way ANOVA).
(TIF)

Figure S3 **Myeloperoxidase (MPO) was measured in BALF of the indicated mouse strains corresponding to the experiments of** **figures 3B** **,** **4F** **, and** **6D** **.** *p<0.05, **p<0.01, ***p<0.001 (1way ANOVA).
(TIF)
