# Supplementary figures and images for: Inflammasome-dependent Pyroptosis and IL-18 Protect against Burkholderia pseudomallei Lung Infection while IL-1β Is Deleterious
Source: PLoS Pathog. 2011 Dec 29;7(12):e1002452. doi: 10.1371/journal.ppat.1002452 (PMC3248555; doi:10.1371/journal.ppat.1002452)

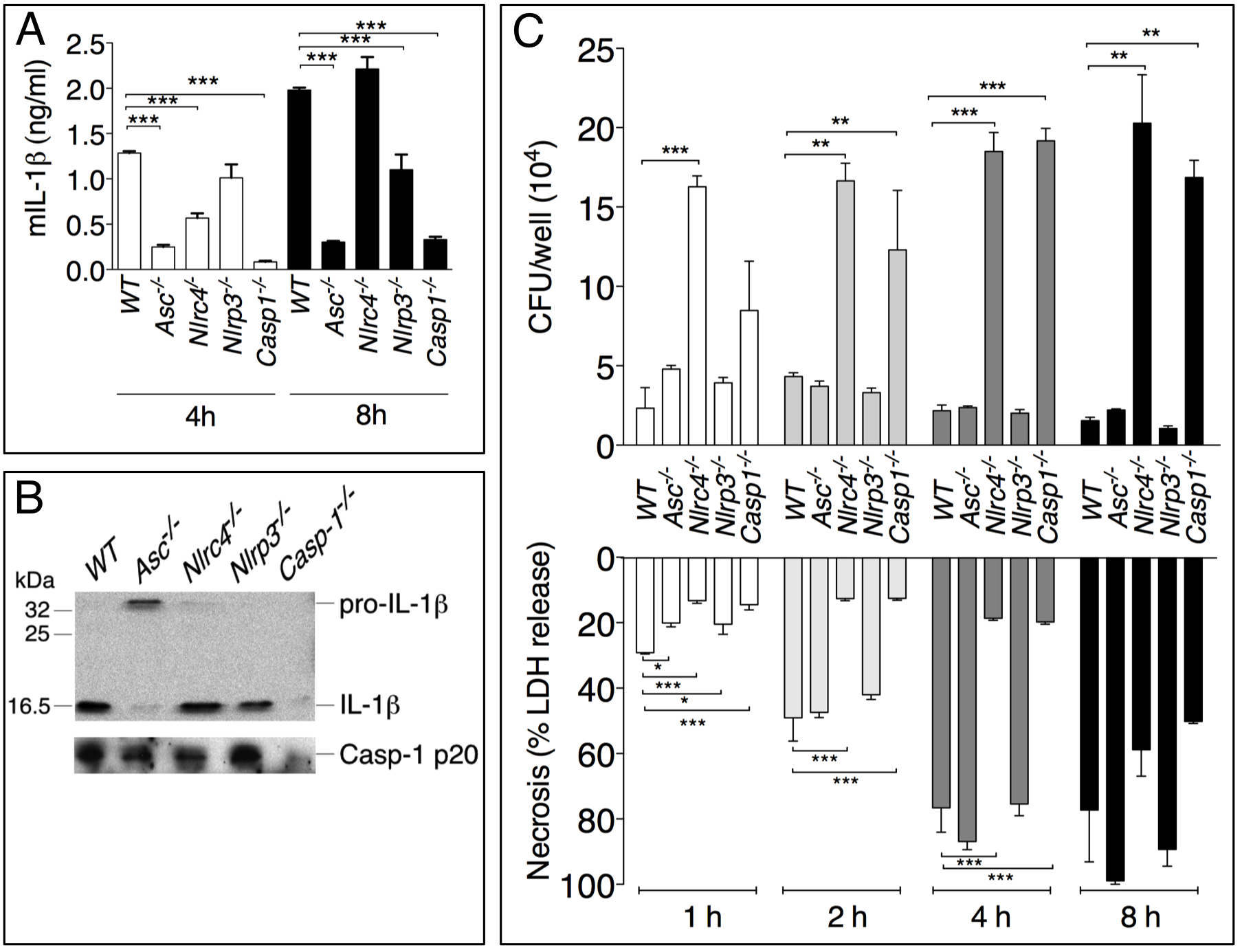

Supplement: Figure S1 — NLRP3 and NLRC4 differentially regulate production of IL-1β and IL-18 and pyroptosis. BMDC were infected with B. pseudomallei at MOI of 10. (A) Secretion of mature IL-1β was measured in conditioned supernatants at the indicated times. (B) Processing of IL-1β and caspase-1 were detected by immunoblot in 8h conditioned supernatants from A. (C) BMDC infected with B. pseudomallei (MOI 10) were lysed at the indicated time points after infection and intracellular bacterial growth was quantitated (upper panel). Induction of pyroptosis was measured as LDH release in conditioned supernatants (lower panel). One experiment representative of four (A) or three (C) is shown. *p<0.05, **p<0.01, ***p<0.001 (1way ANOVA). (TIF) [file ppat.1002452.s001.tif]

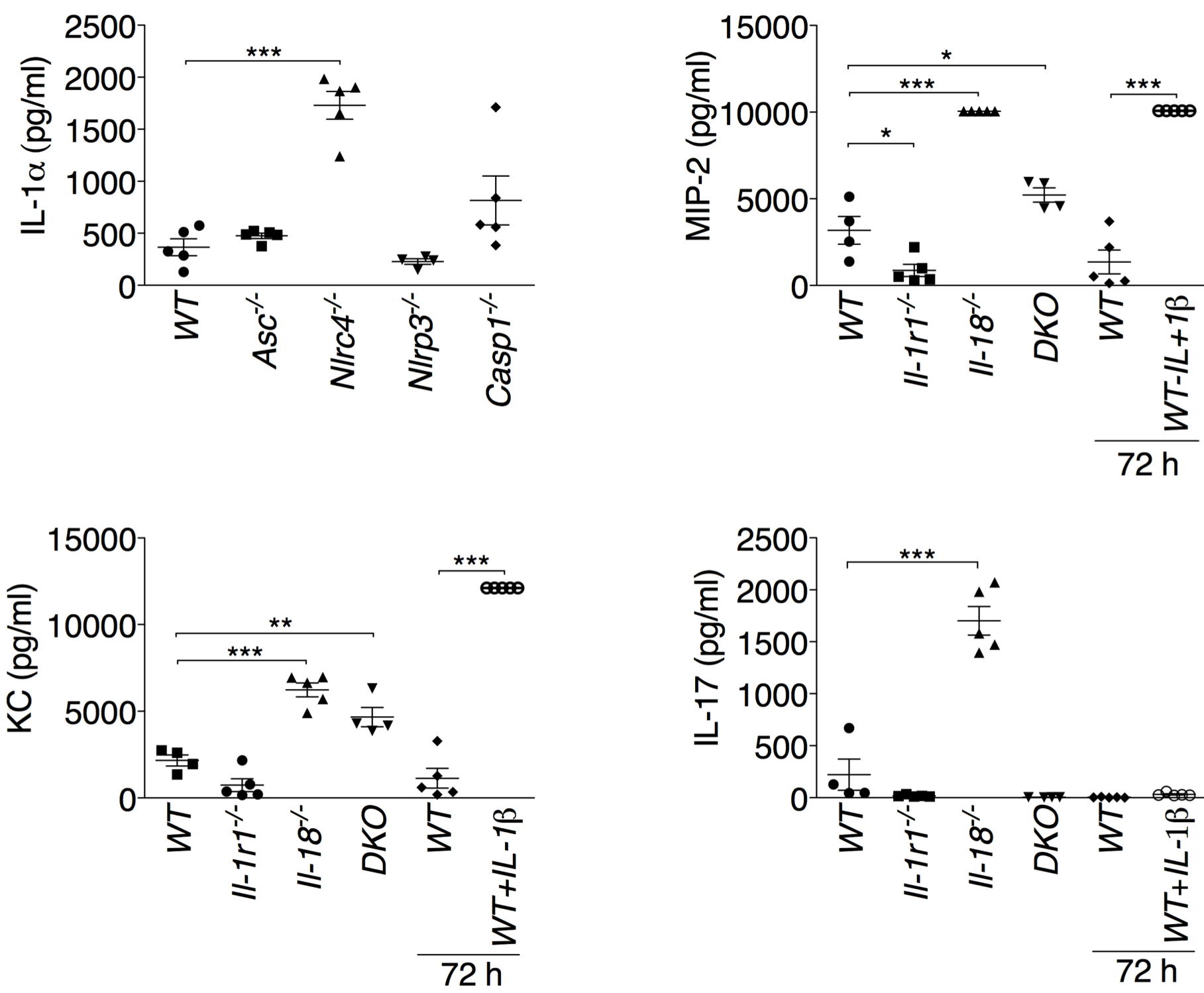

Supplement: Figure S2 — Cytokines and chemokines were measured in BALF obtained from the indicated mouse strains 48 hours or 72 hours post-infection, as shown. *p<0.05, **p<0.01, ***p<0.001 (1way ANOVA). (TIF) [file ppat.1002452.s002.tif]

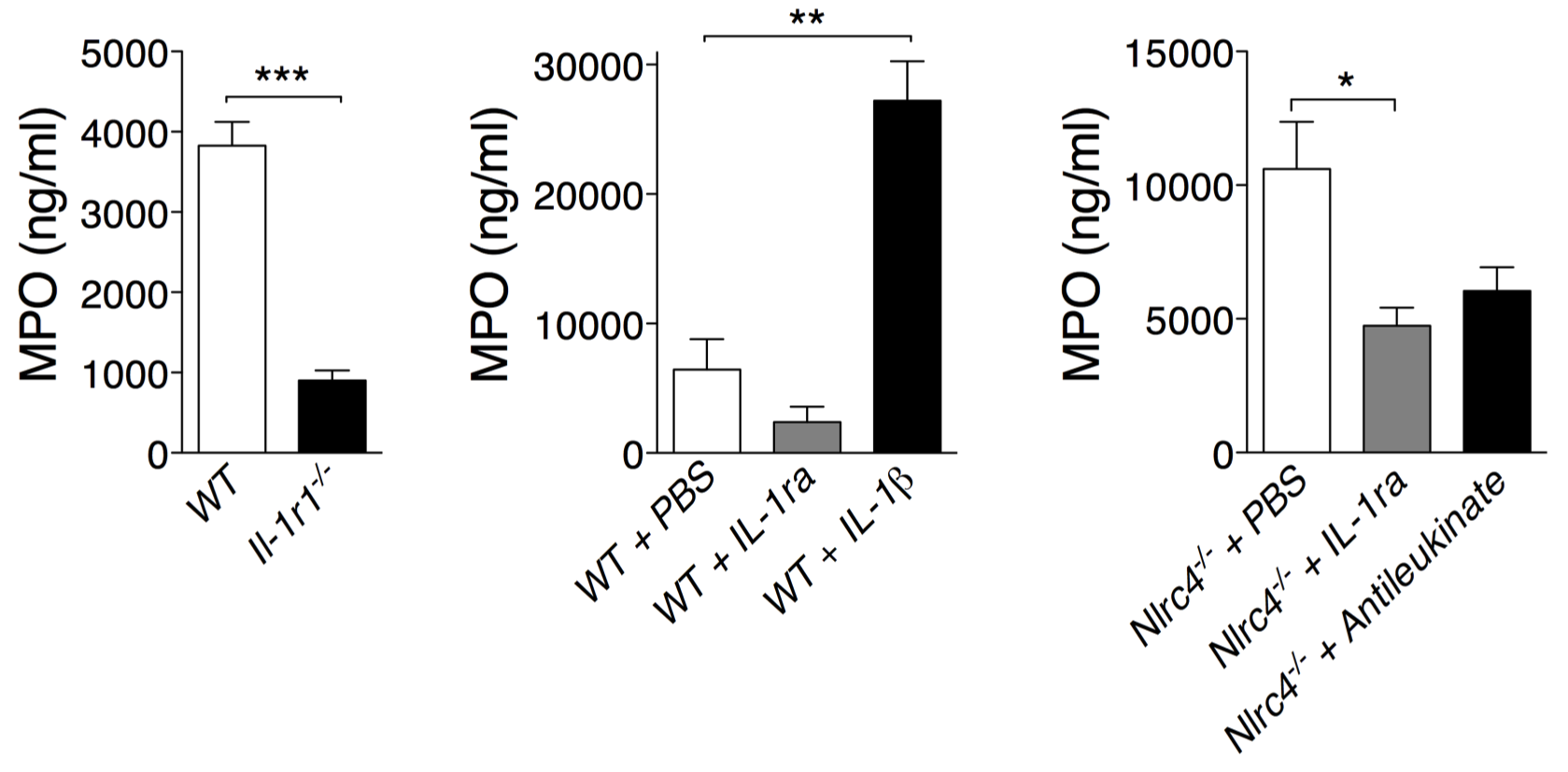

Supplement: Figure S3 — Myeloperoxidase (MPO) was measured in BALF of the indicated mouse strains corresponding to the experiments of figures 3B , 4F , and 6D . *p<0.05, **p<0.01, ***p<0.001 (1way ANOVA). (TIF) [file ppat.1002452.s003.tif]
